# Inhibition of PDGFR signaling prevents muscular fatty infiltration after rotator cuff tear in mice

**DOI:** 10.1038/srep41552

**Published:** 2017-01-31

**Authors:** Hideyuki Shirasawa, Noboru Matsumura, Masayuki Shimoda, Satoshi Oki, Masaki Yoda, Takahide Tohmonda, Yae Kanai, Morio Matsumoto, Masaya Nakamura, Keisuke Horiuchi

**Affiliations:** 1 Department of Orthopedic Surgery, and Keio University School of Medicine, Tokyo 160-8582, Japan; 2 Department of Pathology, Keio University School of Medicine, Tokyo 160-8582, Japan.

## Abstract

Fatty infiltration in muscle is often observed in patients with sizable rotator cuff tear (RCT) and is thought to be an irreversible event that significantly compromises muscle plasticity and contraction strength. These changes in the mechanical properties of the affected muscle render surgical repair of RCT highly formidable. Therefore, it is important to learn more about the pathology of fatty infiltration to prevent this undesired condition. In the present study, we aimed to generate a mouse model that can reliably recapitulate some of the important characteristics of muscular fatty infiltration after RCT in humans. We found that fatty infiltration can be efficiently induced by a combination of the following procedures: denervation of the suprascapular nerve, transection of the rotator cuff tendon, and resection of the humeral head. Using this model, we found that platelet-derived growth factor receptor-α (PDGFRα)-positive mesenchymal stem cells are induced after this intervention and that inhibition of PDGFR signaling by imatinib treatment can significantly suppress fatty infiltration. Taken together, the present study presents a reliable fatty infiltration mouse model and suggests a key role for PDGFRα-positive mesenchymal stem cells in the process of fatty infiltration after RCT in humans.

Rotator cuff tear (RCT) is one of the most common ailments observed in orthopedic patients. Although approximately half of the cases with RCT are asymptomatic^1^, RCT often results in persistent pain and severe functional defects in the affected shoulder joint^2^,^3^. Because RCT most frequently affects the elderly population, patient numbers are markedly increasing in aging societies. Therefore, it is necessary to learn more about the pathology of this disorder and to establish a treatment modality for these patients.

Currently, surgical repair of the ruptured tendons is considered to be one of the preferred treatments for patients with RCT^2^,^4^. However, the surgical repair is often time intensive and results in re-tearing of the repaired tendon^5^. There are various factors, including age^6^,^7^,^8^,^9^,^10^,^11^, the size and extent of the tear retraction^7^,^9^,^10^,^11^, duration of symptom^12^, and degeneration of the damaged tissue^7^,^9^,^11^,^13^, that potentially contribute to the poor clinical outcome of surgical treatment. Among these factors, degenerative changes in the rotator cuff muscle are reported to be one of the most critical determinants^6^,^7^,^11^,^13^,^14^. After RCT, the corresponding muscle retracts and undergoes atrophy, which is accompanied by an increase in intramuscular fat^15^,^16^,^17^. This phenomenon, termed fatty infiltration or fatty degeneration, is an irreversible event and significantly compromises muscle plasticity and contraction strength^14^. These changes in the muscle not only make the surgical repair of the tendon difficult but also increase the risk of re-tear^13^. Therefore, development of a therapeutic intervention to prevent fatty infiltration is desirable to improve the surgical outcome.

Recent studies have shown the presence of stem cells in the skeletal muscle that are distinct from satellite cells. These cells are identified as platelet-derived growth factor receptor-α-positive mesenchymal stem cells (PDGFRα^+^ MSCs) or fibro/adipogenic progenitors (FAPs) (hereafter referred to as PDGFRα^+^ MSCs for consistency)^18^,^19^,^20^. Although not proven, published data suggest that these two cell populations (FAPs and PDGFRα^+^ MSCs) are closely related, if not identical^21^. Unlike satellite cells, which specifically differentiate into myoblasts and muscle fiber cells, PDGFRα^+^ MSCs have the potential to differentiate into osteoblasts, fibroblasts and adipocyte lineages *in vivo* and *in vitro* but lack the capacity to differentiate into myoblast lineages^22^,^23^,^24^,^25^. These studies indicate that the adipocyte progenitors responsible for fatty infiltration after massive RCT are derived from PDGFRα^+^ MSCs and that these cells could constitute a potential target in preventing fatty infiltration. However, this hypothesis has not been fully addressed.

In this study, we describe a mouse model that can efficiently induce fatty infiltration in the suprascapular (SSP) muscle in 4 weeks. Furthermore, using this mouse model, we found that PDGFRα-positive cells are induced after massive RCT and that pharmacological inhibition of PDGFR can significantly suppress the progression of fatty infiltration. Therefore, our data suggest that PDGFR is a potential molecular target for preventing fatty infiltration after massive RCT.

## Methods

### Generation of a fatty infiltration mouse model

**Figure 1:**
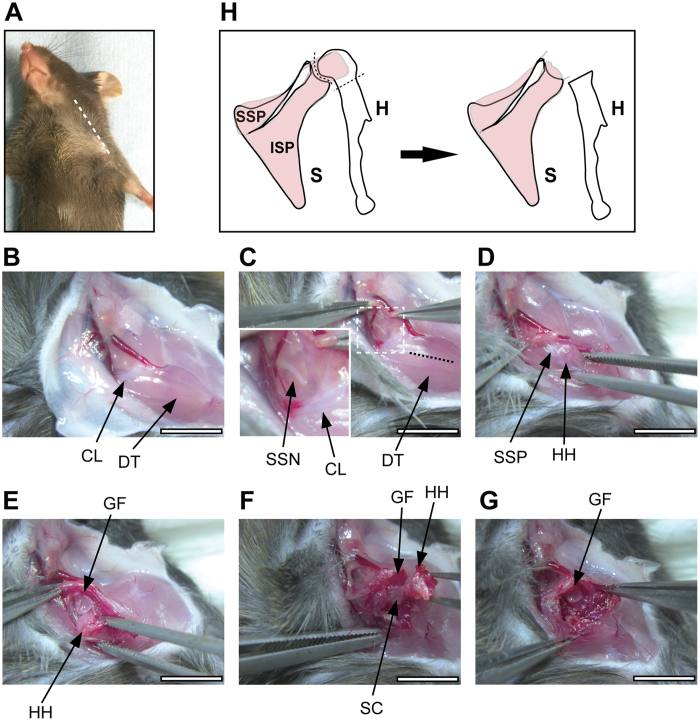
The generation of a fatty infiltration mouse model. (**A**–**G**) Macroscopic images highlighting the outline of the procedure. The dotted line indicates the skin incision (**A**). The inset shows the magnification of the boxed area (**C**). (**H**) A schematic of the present procedure. CL, clavicle bone; DT, deltoid muscle; SSN, suprascapular nerve; SSP, supraspinatus tendon; HH, humeral head; GF, glenoid fossa; SSC, subscapularis tendon; ISP, infraspinatus tendon; S, scapula, H, humerus. Bar, 5 mm.

All of the animal experiments were approved by the Institutional Animal Care and Use Committee at the Keio University School of Medicine (approval number 11022). All methods were performed in accordance with the guidelines and regulations. C57BL/6 male mice (Clea Japan, Tokyo, Japan; 9 weeks old; body weight, approx. 23 g) were used in the present study. The surgical procedures were performed under general anesthesia with an intraperitoneal injection of a mixture of sterilized water, medetomidine (30 μg/ml), midazolam (400 μg/ml), and butorphanol (500 μg/ml) at a volume of 0.1 ml per 10 g body weight. The surgical interventions in the rotator cuff were performed in the left shoulder of the mice, whereas a sham surgery was performed in the right shoulder. Denervation and rotator cuff tendon transection were performed as previously described with some modifications^26^,^27^. Mice were placed on a surgical table, and the clavicle bone and the deltoid muscle were exposed through a skin incision (Fig. 1A and B). The suprascapular nerve was carefully exposed at the supraclavicular fossa (Fig. 1C), and a segment of the nerve at least 5 mm long was resected. The deltoid muscle was longitudinally split to expose the SSP tendon (Fig. 1D). The rotator cuff (including the SSP tendon, infraspinatus tendon, teres minor tendon, and subscapularis tendon) and the long head of the biceps were circumferentially transected (Fig. 1E). Furthermore, the humerus head was removed to prevent the reattachment of the transected tendons (Fig. 1F and G). The skin incision was closed using a 5-0 nylon suture. For the sham surgery in the right shoulder, the same skin and muscle incisions were performed, but the rotator cuff and suprascapular nerve were left untouched. The right shoulder specimens were used as controls (Ctrl) in the present study. At the time of analysis, the shoulder girdle and the upper limb were excised en bloc with the muscles and connective tissues. The specimens were examined under a dissecting microscope to evaluate if the severed tendons were reattached to the humerus or other structures that can lead to load transmission.

### Antibodies

The following antibodies were used in the present study: anti-GAPDH (1:1000; Sigma-Aldrich, Saint Louis, MO), anti-Laminin 2 (1:500; 4H8-2, Sigma-Aldrich), anti-PDGFRα (1:50; Santa Cruz Biotechnology, Dallas, TX), and anti-Perilipin (1:500; D1D8, Cell Signaling Technology, Danvers, MA). Alexa Fluor 488- and Alexa Fluor 546-conjugated anti-rabbit and anti-rat IgG antibodies were acquired from Life technologies (Carlsbad, CA).

### Quantitative PCR

The resected muscles were weighted and lysed using Sepasol RNA I Super G (Nacalai Tesque, Kyoto, Japan) and a bead crusher (μT-12, Taitec, Saitama, Japan). PCR amplification and quantification were performed using the THUNDERBIRD SYBR qPCR Mix (Toyobo, Osaka, Japan) and a 7300 Real-Time PCR System (Applied Biosystems, Foster City, CA). The relative mRNA expression levels were normalized to *Gapdh* expression levels. The expression level of a given transcript in the Ctrl shoulders was set to 1 to calculate changes in the expression levels of muscles from the surgically altered shoulders. The nucleotide sequences of the oligos used in the present study are shown in Table 1. Quantitative PCR was performed in triplicate for each specimen.

### Histology and immunostaining

The muscle tissues were fixed in 4% paraformaldehyde (in phosphate-buffered saline [PBS]) overnight, embedded in paraffin, and cut into 4-μm-thick sections. For the immunostaining of PDGFRα, frozen sections were used. The muscle tissues were mounted on cork disks with tragacanth gum and frozen by immersion in a liquid nitrogen-cooled isopentane bath. The frozen specimens were sectioned at a 7-μm thickness using a cryostat (CM3050 S, Leica Biosystems, Nussloch, Germany). The sections were incubated with blocking solution (Blocking One, Nacalai) for 60 min and subsequently treated with the appropriate primary antibody diluted in blocking solution at 4 degrees overnight. After several washes, the sections were incubated with secondary antibody for 90 min. After several washes, the sections were incubated with secondary antibody for 30 min. The slides were washed several times and mounted with cover slips using VECTASHIELD HardSet Antifade Mounting Medium with DAPI (Vector Laboratories, Burlingame, CA). Fluorescent images were acquired using an FSX100 Inverted Microscope (Olympus, Tokyo, Japan) and an FV10i confocal laser scanning microscope (Olympus). The contrast of the images was adjusted using Adobe Photoshop CC (San Jose, CA).

### Image analysis

The sections sliced from the muscle belly were used for image analysis. Digital images of the sections were acquired as described above. Areas of adipocytes/fatty tissues and the number of PDGFRα cells/section were manually examined and quantified using ImageJ software ( https://imagej.nih.gov/ij/). The values for intraclass correlation coefficient (1, 1) were 0.951 (95% confidence interval; 0.804–0.994) and 0.963 (95% confidence interval; 0.847–0.996) for sections stained for Perilipin (n = 5 sections) and PDGFRα (n = 5 sections), respectively.

### Statistical analysis

**Figure 2:**
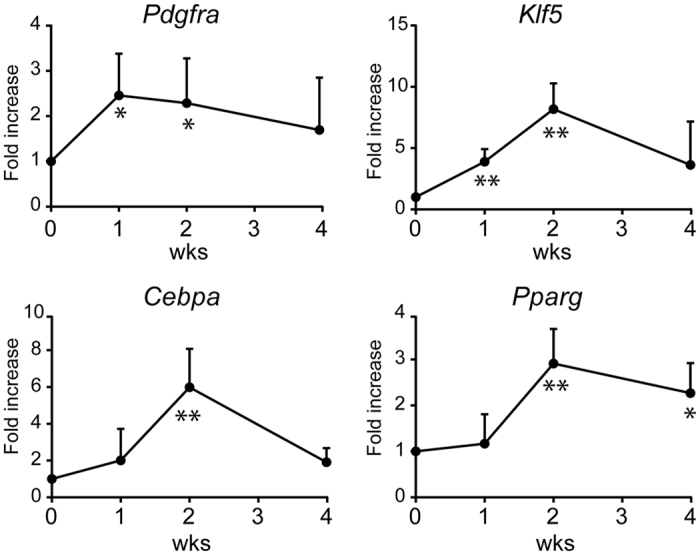
Time course analysis of the transcripts for *Pdgfra* and adipocyte markers. wks, weeks. The values represent the means ± S.D. n = 5 mice/each time point. Quantitative PCR was performed in triplicate. *p < 0.05; **p < 0.01.

**Figure 3:**
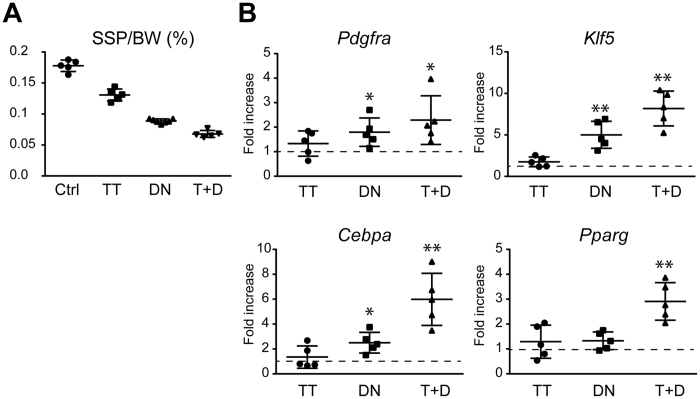
The transcripts for *Pdgfra* and the adipocyte markers are highly induced in the SSP muscle after denervation and rotator cuff tendon transection. (**A**) The proportional weight of the SSP muscle to the body weight 2 weeks after the intervention. The values represent the means ± S.D. n = 5 mice/group. The values of each group were significantly different from one another (ANOVA, p < 0.001). (**B**) Expression levels of the transcripts for *Pdgfra* and the adipocyte markers. The values represent the means ± S.D. of the fold increase of each transcript over the basal expression level (dotted line). n = 5 mice/group. Quantitative PCR was performed in triplicate. *p < 0.05; **p < 0.01.

**Figure 4:**
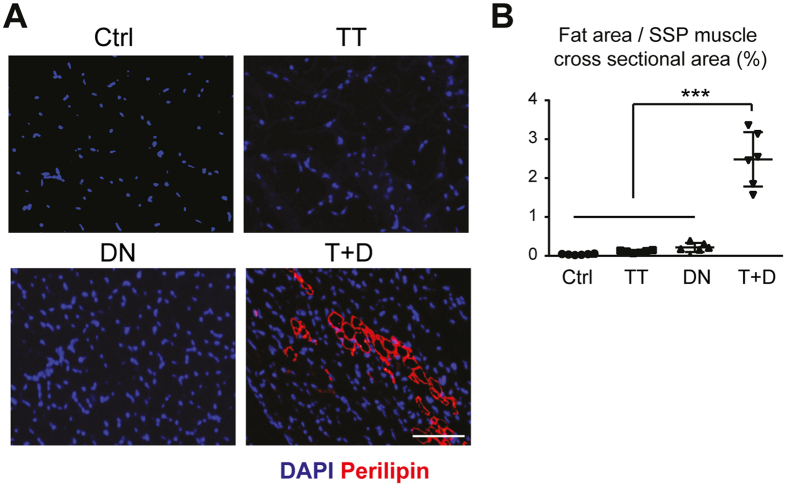
The combination procedure of denervation and rotator cuff transection leads to fatty infiltration in the SSP muscle. (**A**) Immunofluorescent images of the SSP muscle sections stained for perilipin and with DAPI. Representative images of 3 biological replicates are shown. Bar, 50 μm. (**B**) The proportional areas of the adipocytes in the SSP muscle sections. The values represent the means ± S.D. n = 3 mice/group. Two different sections were evaluated in each muscle specimen. ***p < 0.001.

**Figure 5:**
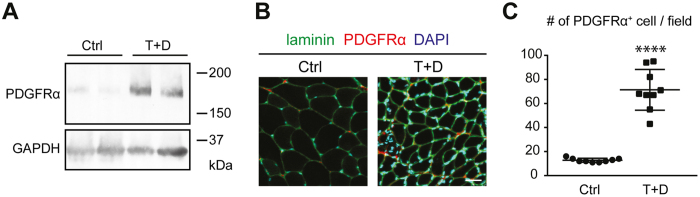
PDGFRα^+^ MSCs are induced in the T + D group. (**A**) The tissue lysates of the SSP muscle collected from surgically treated and sham-operated specimens (2 biological replicates) were subjected to Western blotting using anti-PDGFRα (upper panel) and anti-GAPDH antibodies (lower panel). (**B**) Immunofluorescent images of the SSP muscle sections stained for laminin and PDGFRα and with DAPI. Representative images of 3 biological replicates are shown. Bar, 30 μm. (**C**) The number of PDGFRα-positive cells per microscopic field (214 μm × 214 μm) of SSP muscle section. The values represent the means ± S.D. n = 3 mice. Three different sections were analyzed in each specimen. ****p < 0.0001.

**Figure 6:**
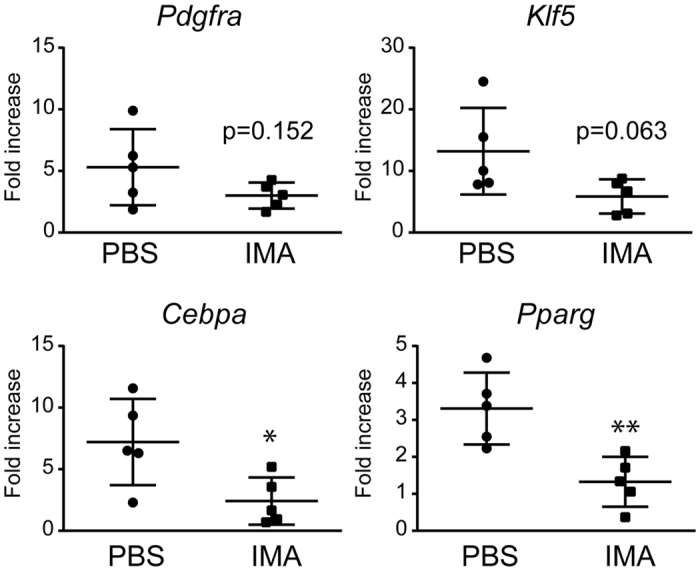
Treatment with imatinib suppresses the expression of adipocyte markers. The expression levels of the transcripts for *Pdgfra* and the adipocyte markers are shown. The values represent the means ± S.D. of the fold increase of each transcript over the basal expression level. n = 5 mice/group. Quantitative PCR was performed in triplicate. *p < 0.05; **p < 0.01.

**Figure 7:**
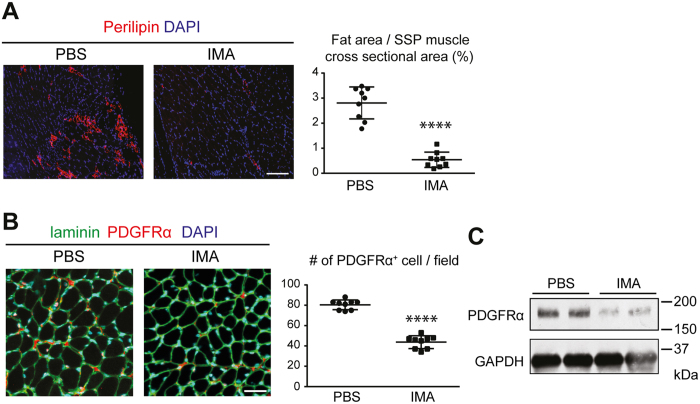
Treatment with imatinib suppresses the development of fatty infiltration in the SSP muscle after denervation and tendon transection. (**A**) Immunofluorescent images of the SSP sections stained for perilipin and with DAPI. Representative images of 3 biological replicates are shown (left panel). Bar, 100 μm. The ratio of the fatty tissue area in the SSP muscle sections is shown (right panel). Three different sections from each individual mouse were analyzed. n = 3 mice/group. ****p < 0.0001. (**B**) Immunofluorescent images of the SSP muscle sections stained for laminin and PDGFRα and with DAPI. Representative images of 3 biological replicates are shown (left panel). Bar, 30 μm. The number of PDGFRα-positive cells per microscopic field (214 μm × 214 μm) in the SSP muscle sections is shown (right panel). Three different sections from each individual mouse were analyzed. n = 3 mice/group. ****p < 0.0001. (**C**) Western blot analysis of the SSP tissues probed for PDGFRα and GAPDH. n = 2 mice/group.

A one-sample t-test (two-tailed) was used for Figs 2 and 3B. One-way ANOVA followed by Tukey’s post-hoc test was used for Figs 3A and 4B. Student’s t-test (two-tailed) was used in Figs 5C,6,7A and B. The significance was set at 0.05 for all analyses. The values are presented as the means ± the standard deviations. All of the statistical analyses were conducted using GraphPad Prism software (Ver. 6.07; La Jolla, CA). The intraclass correlation coefficient was analyzed using SPSS 22 (IBM, Armonk, NY).

## Results

### Transcripts for adipocyte markers are induced following denervation and tendon transection

To characterize the biological changes in the SSP muscle after surgical intervention, we first examined the transcript expression levels of genes with crucial roles in adipocyte differentiation, including *Klf5, Cebpa*, and *Pparg*^28^,^29^. A total of 15 mice underwent the surgical intervention consisting of denervation of the suprascapular nerve and rotator cuff transection as described in the Methods. Five mice were analyzed at each time point (1, 2, and 4 weeks). All of the mice tolerated the procedure over the designated time period without any major issues or complications. At the time of analysis, we confirmed that there was apparently no tendon reattachment or scar tissue formation, which could potentially reconnect the transected SSP tendon to the humerus. Time course analysis of the gene expression pattern showed that all of the transcripts for these genes are transiently induced at approximately 2 weeks after the intervention (Fig. 2). Interestingly, we also found that the expression of the transcripts for *Pdgfra*, a marker for PDGFRα^+^ MSCs in the skeletal muscle^18^,^19^, peaked approximately 1 week after the intervention, which preceded the expression of the adipocyte markers. Based on these findings, the expression levels of the transcripts were analyzed 2 weeks after surgical intervention in the subsequent experiments.

### Combined intervention of denervation and rotator cuff transection is required to promote intramuscular fatty infiltration

We next asked whether rotator cuff transection or denervation alone is sufficient to induce fatty infiltration in the SSP muscle. A total of 24 mice were randomly divided into 3 groups (8 mice/group): the rotator cuff tendon transection group (TT; rotator cuff transection and resection of the humeral head), the denervation of the suprascapular nerve group (DN), and the tendon transection and denervation group (T + D). There were no statistically significant differences in body weight among the groups (data not shown). Gene expression analysis and muscle weight were analyzed 2 weeks after surgery (5 mice/group), and histological analyses were performed 4 weeks after surgery (3 mice/group).

Both the TT and DN groups showed significant decreases in the proportional weight of the SSP muscle compared to the Ctrl group. The combination of denervation and tendon transection (T + D group) showed an addictive effect on muscle atrophy (Fig. 3A). The expression levels of the transcripts for the adipocyte markers and *Pdgfra* were higher in the group receiving the combined intervention of denervation and tendon transection (T + D) compared to the groups receiving either one of the interventions (Fig. 3B). Immunostaining of the SSP muscle sections for perilipin, a membrane-bound protein specifically expressed in adipocytes, showed a marked increase in the number of adipocytes only in the T + D group (Fig. 4A). In accordance, histomorphometric analysis of the fatty cell area was significantly increased in the T + D group compared to the other 2 groups and the Ctrl (Fig. 4B). These observations suggest that, although either denervation or tendon transection alone is sufficient to induce muscle atrophy, the combination of both interventions is required to efficiently promote fatty infiltration in the present mouse model.

### The population of PDGFRα-positive cells increases after the combined procedure

The rapid increase in the expression of *Pdgfra* transcripts after the intervention (Fig. 2) indicates that the population of PDGFRα^+^ MSCs rapidly expands after denervation and tendon transection in the SSP muscle. Using the extracts of the SSP muscle tissues, Western blot analysis showed a marked increase of PDGFRα protein in the T + D group compared to the Ctrl group (Fig. 5A). Concomitantly, immunostaining for PDGFRα showed a significant increase in the number of PDGFRα^+^ cells in the SSP muscle tissues collected from the T + D group compared to the Ctrl group (Fig. 5B and C). These results indicate that the combined procedure of denervation and tendon transection enhances the proliferation of PDGFRα^+^ MSCs, which subsequently differentiate into adipocytes in the SSP muscle.

### Suppression of PDGFRα signaling by imatinib attenuates fatty infiltration

Because PDGFRα^+^ MSCs are the major source of adipocytes in skeletal muscle^18^ and the population of these cells increases after denervation and tendon transection, we hypothesized that suppression of PDGFRα^+^ MSC proliferation would attenuate fatty infiltration in the SSP muscle. To this end, mice treated with both denervation and tendon transection were randomly divided into two groups (13 mice/group) and were orally administered either imatinib mesylate (30 mg/kg, 5 days/week), a potent PDGFR inhibitor^30^, or PBS. Gene expression analysis was performed 2 weeks after the surgery, and histological analysis and Western blot assay were performed 4 weeks after the surgery. Gene expression analysis showed significant decreases in the transcript levels of the late-stage adipocyte markers *Cebpa* and *Pparg* in the imatinib-treated group compared to the PBS-treated control group (Fig. 6). However, at the same time point, the differences in the transcript levels for the early differentiation markers *Pdgfra* and *Klf5* were not statistically significant between the two groups.

Histological analysis revealed significant decreases in the numbers of both perilipin-positive cells and PDGFRα-positive cells in mice treated with imatinib compared to PBS-treated controls (Fig. 7A and B). The decrease in the expression of PDGFRα in the SSP muscle collected from imatinib treated-mice was confirmed by Western blot (Fig. 7C). These observations indicate that the decrease in PDGFRα-positive cells is correlated with the suppression of fatty infiltration after surgical intervention.

## Discussion

The present study describes a reliable mouse model which can efficiently induce fatty infiltration in the SSP muscle in 4 weeks. Using this model, we found that PDGFRα-positive cells are rapidly induced after surgical intervention and that imatinib administration markedly suppresses fatty infiltration, suggesting that inhibition of the proliferation PDGFRα^+^ MSCs may potentially ameliorate fatty infiltration in the SSP muscle after RCT in humans.

Our fatty infiltration mouse model is in accordance with previously reported RCT models in that a combined procedure of denervation of the suprascapular nerve and rotator cuff tendon transection is required to efficiently induce fatty infiltration^26^,^27^,^31^. On the other hand, there are some differences between the current and previously reported models. In one mouse model, the SSP and infraspinatus tendons were transected, and the subscapularis and teres minor tendons were left untouched^26^. However, in our preliminary experiments, we often observed spontaneous reattachment of the tendons to the humerus in this mouse model. We hypothesized that any tensile stress to the muscle could potentially suppress the development of fat infiltration, and complete transection of all four tendons of the rotator cuff and removal of the humeral head were necessary to prevent the reattachment of the tendons to the humerus. We analyzed more than 20 mice at 4 weeks after the procedure and found no regenerated tendons or scar tissue that could possibly reattach the SSP muscle to the humerus. In previous studies, histological analysis was performed 12 weeks after surgery, and a marked increase in the intramuscular fat was observed at this time point^26^,^32^. However, the increase in the transcripts for adipocyte markers was observed as early as 2 weeks after the surgery, and fatty infiltration was obvious even at 4 weeks after the surgical intervention in the present model, indicating that suppression of the mechanical load to the muscle could enhance this process. Accordingly, studies have shown the crucial involvement of mechanical stress in regulating adipocyte differentiation^33^,^34^,^35^. A study is currently underway to elucidate whether adipocyte differentiation of PDGFRα^+^ MSCs is affected by mechanical stress in skeletal muscle. Nevertheless, the present study suggests that loss of tensile stress in the muscle is causally related to the development of fatty infiltration.

We found that after the combined intervention of denervation and tendon transection, the proliferation of PDGFRα-positive cells and the increase in the transcript levels of *Pdgfra* precedes fatty infiltration and the increases in adipocyte marker transcript levels. These observations indicate that a complete loss of mechanical stress triggers the proliferation of PDGFRα^+^ MSCs and that expansion of these cells is related to subsequent fatty infiltration in the SSP muscle. Most importantly, our data also suggest that inhibition of PDGFR signaling by imatinib treatment can suppress PDGFRα^+^ MSC proliferation and the ensuing fatty infiltration in the present model. In accordance with this hypothesis, a recent study showed that inhibition of TGF-β1 reduces the number of PDGFRα^+^ MSCs, thereby suppressing the progression of fatty infiltration after RCT in a mouse model^32^.

Although these observations promote the idea that PDGFRα^+^ MSCs are the source of adipocytes after RCT, there still are other possibilities. The intramuscular fat can potentially be explained by the proliferation of pre-existing adipocytes, the infiltration/invasion of adipocytes surrounding the muscle, or the differentiation of pluripotent stem cells that are not related to PDGFRα^+^ MSCs^36^. Although the present study does not provide a conclusive answer to these issues, the data show that proliferation and adipocytic differentiation of PDGFRα^+^ MSCs are, at least in part, responsible for this phenomenon. In addition, our data also suggest that a loss of muscle tensile stress, but not denervation or tendon injury *per se*, is responsible for triggering the proliferation and differentiation of quiescent PDGFRα^+^ MSCs residing in the muscle. However, the molecular mechanisms involved in the activation of PDGFRα^+^ MSCs and the preferential differentiation of PDGFRα^+^ MSCs towards adipocytes after massive RCT are still unknown.

We would like to mention herein that, considering PDGFRα^+^ MSCs residing in the muscle as the major source of adipocytes, the use of the term “fatty infiltration” or “fatty degeneration” may not be scientifically sound. Although we have used “fatty infiltration” in the present manuscript for consistency and convenience, “fatty proliferative change” would be more appropriate terminology to depict this condition. Clinically, fatty infiltration has negative impacts on muscle function and mechanical properties; however, this phenomenon can be recapitulated in several different mammal models and is preserved over different species^26^,^27^,^31^,^37^,^38^. Given that fundamental biological functions are generally conserved beyond species, it would be of interest to explore whether fatty infiltration has beneficial effects on metabolism or systematic homeostasis.

There are several limitations to the present study. First, as with other types of animal models, the differences in the size, anatomy, and physiology compared to those of humans can never be overlooked. Second, we primarily used relatively young (i.e., 9-week-old) mice in the present study, and it is unclear how aging would affect the development of fatty infiltration. Because patients with RCT and fatty infiltration are mostly among the elderly population, the results of animal model studies, which often use relatively young subjects, should be interpreted with caution. Third, although the excision of the humeral head was effective in minimizing the chance of reattachment of the severed tendons to the humerus, the trauma caused by this procedure can potentially have some effects on fatty infiltration or muscle atrophy that do not necessarily reflect RCT pathology. In addition, because the excision of the humeral head itself dysfunctions the rotator cuff, we could not prepare an adequate sham control to evaluate the impact of this procedure (excision of the humeral head) on fatty infiltration. Nevertheless, we found that the present method is technically simple and yields consistent results in inducing fatty infiltration compared to the conventional methods that do not involve the excision of the humeral head. Fourth, as mentioned above, although our data indicate that inhibition of PDGFR signaling suppresses fatty infiltration, further studies are required to elucidate whether the PDGFRα^+^ MSCs or other types of cells are the source of these intramuscular adipocytes and to determine the direct or indirect involvement of these cells in this process. Conditional abrogation of PDGFRα^+^ MSCs and fate mapping of PDGFRα-positive cells after RCT may help to clarify these issues.

In summary, the present study describes a reliable mouse model that can mimic the histological and biological changes of fatty infiltration after massive RCT in humans. Our data suggest that PDGFRα^+^ MSCs are a potential cellular target for preventing intramuscular fatty infiltration. Although further studies are warranted, the present study may provide novel insight into the pathology and potential treatment modality of fatty infiltration accompanying RCT, to which no effective treatment or prophylaxis currently exists^2^,^4^.

## Additional Information

**How to cite this article:** Shirasawa, H. *et al*. Inhibition of PDGFR signaling prevents muscular fatty infiltration after rotator cuff tear in mice. *Sci. Rep.* **7**, 41552; doi: 10.1038/srep41552 (2017).

**Publisher's note:** Springer Nature remains neutral with regard to jurisdictional claims in published maps and institutional affiliations.

## Figures and Tables

**Table 1:** List of the nucleotide primers used in the present study.

Gene	Strand	Sequence
*Gapdh*	Fwd	TCAACAGCAACTCCCACTCTTCCA
	Rev	ACCCTGTTGCTGTAGCCGTATTCA
*Pdgfra*	Fwd	CTCTTCCCTTCCCACCATTAAC
	Rev	CTCCCGTCAGACCTTGTAATTC
*Cebpa*	Fwd	GGTTTCCTGGGTGAGTTCAT
	Rev	AACCTAGGTCTCTGTCTCCTAC
*Klf5*	Fwd	GGCTCTCCCCGAGTTCACTA
	Rev	ATTACTGCCGTCTGGTTTGTC
*Pparg*	Fwd	CTGGCCTCCCTGATGAATAAAG
	Rev	AGGCTCCATAAAGTCACCAAAG

## References

[b1] Yamamoto A. . Prevalence and risk factors of a rotator cuff tear in the general population. J Shoulder Elbow Surg 19, 116–120 (2010). 19540777 10.1016/j.jse.2009.04.006

[b2] Greenspoon J. A., Petri M., Warth R. J. & Millett P. J. Massive rotator cuff tears: pathomechanics, current treatment options, and clinical outcomes. J Shoulder Elbow Surg 24, 1493–1505 (2015). 26129871 10.1016/j.jse.2015.04.005

[b3] Yamaguchi K. . The demographic and morphological features of rotator cuff disease. A comparison of asymptomatic and symptomatic shoulders. J Bone Joint Surg Am 88, 1699–1704 (2006). 16882890 10.2106/JBJS.E.00835

[b4] Gerber C., Fuchs B. & Hodler J. The results of repair of massive tears of the rotator cuff. J Bone Joint Surg Am 82, 505–515 (2000). 10761941 10.2106/00004623-200004000-00006

[b5] Sugaya H., Maeda K., Matsuki K. & Moriishi J. Repair integrity and functional outcome after arthroscopic double-row rotator cuff repair. A prospective outcome study. J Bone Joint Surg Am 89, 953–960 (2007). 17473131 10.2106/JBJS.F.00512

[b6] Liem D., Lichtenberg S., Magosch P. & Habermeyer P. Magnetic resonance imaging of arthroscopic supraspinatus tendon repair. J Bone Joint Surg Am 89, 1770–1776 (2007). 17671017 10.2106/JBJS.F.00749

[b7] Oh J. H., Kim S. H., Kang J. Y., Oh C. H. & Gong H. S. Effect of age on functional and structural outcome after rotator cuff repair. Am J Sports Med 38, 672–678 (2010). 20357401 10.1177/0363546509352460

[b8] Voigt C., Bosse C., Vosshenrich R., Schulz A. P. & Lill H. Arthroscopic supraspinatus tendon repair with suture-bridging technique: functional outcome and magnetic resonance imaging. Am J Sports Med 38, 983–991 (2010). 20436053 10.1177/0363546509359063

[b9] Nho S. J. . Prospective analysis of arthroscopic rotator cuff repair: subgroup analysis. J Shoulder Elbow Surg 18, 697–704 (2009). 19269861 10.1016/j.jse.2008.11.018

[b10] Tashjian R. Z. . Factors affecting healing rates after arthroscopic double-row rotator cuff repair. Am J Sports Med 38, 2435–2442 (2010). 21030564 10.1177/0363546510382835

[b11] Chung S. W., Oh J. H., Gong H. S., Kim J. Y. & Kim S. H. Factors affecting rotator cuff healing after arthroscopic repair: osteoporosis as one of the independent risk factors. Am J Sports Med 39, 2099–2107 (2011). 21813440 10.1177/0363546511415659

[b12] Charousset C., Bellaiche L., Kalra K. & Petrover D. Arthroscopic repair of full-thickness rotator cuff tears: is there tendon healing in patients aged 65 years or older? Arthroscopy 26, 302–309 (2010). 20206038 10.1016/j.arthro.2009.08.027

[b13] Goutallier D., Postel J. M., Bernageau J., Lavau L. & Voisin M. C. Fatty muscle degeneration in cuff ruptures. Pre- and postoperative evaluation by CT scan. Clin Orthop Relat Res, 78–83 (1994). 8020238

[b14] Gladstone J. N., Bishop J. Y., Lo I. K. & Flatow E. L. Fatty infiltration and atrophy of the rotator cuff do not improve after rotator cuff repair and correlate with poor functional outcome. Am J Sports Med 35, 719–728 (2007). 17337727 10.1177/0363546506297539

[b15] Gerber C., Schneeberger A. G., Hoppeler H. & Meyer D. C. Correlation of atrophy and fatty infiltration on strength and integrity of rotator cuff repairs: a study in thirteen patients. J Shoulder Elbow Surg 16, 691–696 (2007). 17931904 10.1016/j.jse.2007.02.122

[b16] Melis B., DeFranco M. J., Chuinard C. & Walch G. Natural history of fatty infiltration and atrophy of the supraspinatus muscle in rotator cuff tears. Clin Orthop Relat Res 468, 1498–1505 (2010). 20094853 10.1007/s11999-009-1207-x PMC2865597

[b17] Fuchs B., Weishaupt D., Zanetti M., Hodler J. & Gerber C. Fatty degeneration of the muscles of the rotator cuff: assessment by computed tomography versus magnetic resonance imaging. J Shoulder Elbow Surg 8, 599–605 (1999). 10633896 10.1016/s1058-2746(99)90097-6

[b18] Uezumi A., Fukada S., Yamamoto N., Takeda S. & Tsuchida K. Mesenchymal progenitors distinct from satellite cells contribute to ectopic fat cell formation in skeletal muscle. Nat Cell Biol 12, 143–152 (2010). 20081842 10.1038/ncb2014

[b19] Joe A. W. . Muscle injury activates resident fibro/adipogenic progenitors that facilitate myogenesis. Nat Cell Biol 12, 153–163 (2010). 20081841 10.1038/ncb2015 PMC4580288

[b20] Uezumi A. . Identification and characterization of PDGFRalpha + mesenchymal progenitors in human skeletal muscle. Cell Death Dis 5, e1186 (2014). 24743741 10.1038/cddis.2014.161 PMC4001314

[b21] Uezumi A., Ikemoto-Uezumi M. & Tsuchida K. Roles of nonmyogenic mesenchymal progenitors in pathogenesis and regeneration of skeletal muscle. Front Physiol 5, 68 (2014). 24605102 10.3389/fphys.2014.00068 PMC3932482

[b22] Yin H., Price F. & Rudnicki M. A. Satellite cells and the muscle stem cell niche. Physiol Rev 93, 23–67 (2013). 23303905 10.1152/physrev.00043.2011 PMC4073943

[b23] Relaix F. & Zammit P. S. Satellite cells are essential for skeletal muscle regeneration: the cell on the edge returns centre stage. Development 139, 2845–2856 (2012). 22833472 10.1242/dev.069088

[b24] Uezumi A. . Fibrosis and adipogenesis originate from a common mesenchymal progenitor in skeletal muscle. J Cell Sci 124, 3654–3664 (2011). 22045730 10.1242/jcs.086629

[b25] Oishi T. . Osteogenic differentiation capacity of human skeletal muscle-derived progenitor cells. PLoS One 8, e56641 (2013). 23457598 10.1371/journal.pone.0056641 PMC3572948

[b26] Liu X. . A mouse model of massive rotator cuff tears. J Bone Joint Surg Am 94, e41 (2012). 22488625 10.2106/JBJS.K.00620

[b27] Kim H. M., Galatz L. M., Lim C., Havlioglu N. & Thomopoulos S. The effect of tear size and nerve injury on rotator cuff muscle fatty degeneration in a rodent animal model. J Shoulder Elbow Surg 21, 847–858 (2012). 21831663 10.1016/j.jse.2011.05.004 PMC3217129

[b28] Cristancho A. G. & Lazar M. A. Forming functional fat: a growing understanding of adipocyte differentiation. Nat Rev Mol Cell Biol 12, 722–734 (2011). 21952300 10.1038/nrm3198 PMC7171550

[b29] Oishi Y. . Kruppel-like transcription factor KLF5 is a key regulator of adipocyte differentiation. Cell Metab 1, 27–39 (2005). 16054042 10.1016/j.cmet.2004.11.005

[b30] Capdeville R., Buchdunger E., Zimmermann J. & Matter A. Glivec (STI571, imatinib), a rationally developed, targeted anticancer drug. Nat Rev Drug Discov 1, 493–502 (2002). 12120256 10.1038/nrd839

[b31] Liu X., Manzano G., Kim H. T. & Feeley B. T. A rat model of massive rotator cuff tears. J Orthop Res 29, 588–595 (2011). 20949443 10.1002/jor.21266

[b32] Davies M. R. . TGF-beta Small Molecule Inhibitor SB431542 Reduces Rotator Cuff Muscle Fibrosis and Fatty Infiltration By Promoting Fibro/Adipogenic Progenitor Apoptosis. PLoS One 11, e0155486 (2016). 27186977 10.1371/journal.pone.0155486 PMC4871364

[b33] Li G. . Mechanical compressive force inhibits adipogenesis of adipose stem cells. Cell Prolif 46, 586–594 (2013). 24033415 10.1111/cpr.12053 PMC6495859

[b34] Sen B. . mTORC2 regulates mechanically induced cytoskeletal reorganization and lineage selection in marrow-derived mesenchymal stem cells. J Bone Miner Res 29, 78–89 (2014). 23821483 10.1002/jbmr.2031 PMC3870029

[b35] Yang X. . Mechanical stretch inhibits adipogenesis and stimulates osteogenesis of adipose stem cells. Cell Prolif 45, 158–166 (2012). 22229452 10.1111/j.1365-2184.2011.00802.x PMC6496762

[b36] Meyer G. A. . Epimuscular Fat in the Human Rotator Cuff Is a Novel Beige Depot. Stem Cells Transl Med 4, 764–774 (2015). 25999520 10.5966/sctm.2014-0287 PMC4479624

[b37] Gupta R. & Lee T. Q. Contributions of the different rabbit models to our understanding of rotator cuff pathology. J Shoulder Elbow Surg 16, S149–157 (2007). 17903710 10.1016/j.jse.2007.05.002

[b38] Gerber C., Meyer D. C., Schneeberger A. G., Hoppeler H. & von Rechenberg B. Effect of tendon release and delayed repair on the structure of the muscles of the rotator cuff: an experimental study in sheep. J Bone Joint Surg Am 86-A, 1973–1982 (2004). 15342760 10.2106/00004623-200409000-00016

